# Mycotoxin Fumonisin B_1_ Interferes Sphingolipid Metabolisms and Neural Tube Closure during Early Embryogenesis in Brown Tsaiya Ducks

**DOI:** 10.3390/toxins13110743

**Published:** 2021-10-20

**Authors:** Chompunut Lumsangkul, Ko-Hua Tso, Yang-Kwang Fan, Hsin-I Chiang, Jyh-Cherng Ju

**Affiliations:** 1 Department of Animal and Aquatic Sciences, Faculty of Agriculture, Chiang Mai University, Chiang Mai 50200, Thailand; chompunut.lum@cmu.ac.th; 2 Science and Technology Research Institute, Chiang Mai University, Chiang Mai 50200, Thailand; 3 Department of Animal Science, National Chung Hsing University, Taichung 40227, Taiwan; d100037004@mail.nchu.edu.tw (K.-H.T.); ykfan@dragon.nchu.edu.tw (Y.-K.F.); 4 Center for the Integrative and Evolutionary Galliformes Genomics, National Chung Hsing University, Taichung 40227, Taiwan; 5 Graduate Institute of Biomedical Sciences, China Medical University, Taichung 40402, Taiwan; 6 Translational Medicine Research Center, China Medical University Hospital, Taichung 40402, Taiwan; 7 Department of Bioinformatics and Medical Engineering, College of Information and Electrical Engineering, Asia University, Taichung 41354, Taiwan

**Keywords:** duck embryos, early embryogenesis, fumonisin B_1_, neural tube defects

## Abstract

Fumonisin B_1_ (FB_1_) is among the most common contaminants produced by *Fusarium* spp. fungus from corns and animal feeds. Although FB_1_ has been known to cause physical or functional defects of embryos in humans and several animal species such as Syrian hamsters, rabbits, and rodents, little is known about the precise toxicity to the embryos and the underlying mechanisms have not been fully addressed. The present study aimed to investigate its developmental toxicity and potential mechanisms of action on sphingolipid metabolism in Brown Tsaiya Ducks (BTDs) embryos. We examined the effect of various FB_1_ dosages (0, 10, 20 and 40 µg/embryo) on BTD embryogenesis 72 h post-incubation. The sphingomyelin content of duck embryos decreased (*p* < 0.05) in the highest FB_1_-treated group (40 µg). Failure of neural tube closure was observed in treated embryos and the expression levels of a neurulation-related gene, sonic hedgehog (*Shh*) was abnormally decreased. The sphingolipid metabolism-related genes including *N*-acylsphingosine amidohydrolase 1 (*ASAH1*), and ceramide synthase 6 (*CERS6*) expressions were altered in the treated embryos compared to those in the control embryos. Apparently, FB_1_ have interfered sphingolipid metabolisms by inhibiting the functions of ceramide synthase and folate transporters. In conclusion, FB_1_-caused developmental retardation and abnormalities, such as neural tube defects in Brown Tsaiya Duck embryos, as well as are partly mediated by the disruption of sphingolipid metabolisms.

## 1. Introduction

Fumonisins (FBs), discovered in 1988, are mycotoxins produced by fungi of the *Fusarium* species including *Fusarium verticillioides* and *Fusarium proliferatum* [1,2]. The most abundant FBs are the B-series, fumonisin B_1_ (FB_1_), B_2_ (FB_2_) and B_3_ (FB_3_), which can be found in natural conditions. The FB_1_ and FB_2_ limitations of the Food and Drug Administration (FDA) in the US [3] and European Union (EU Regulation 1126/2007) [4] were 800–4000 and 2000–4000 μg/kg for cereal-based products, respectively. The average FB_1_ concentration of food exceeds these mycotoxin limits in some countries such as Lebanon and Brazil [5,6]. Among them, FB_1_ is the most toxic and over 70% of food products are contaminated by this toxin [7,8,9], some of which might reach the maximal concentration limit and can be harmful to animal and human health [10].

Previous studies have shown that the consumption of FB_1_ contaminated corn has been associated with esophageal cancer in humans [11,12,13] and with various animal diseases including equine leucoencephalomalacia and hepatotoxicity, as well as porcine pulmonary edema [14,15,16]. FB_1_ has been indicated being associated with the toxicity in different organs, such as livers, kidneys, lungs, and intestine [17,18,19] as well as in nervous and cardiovascular systems of animals [20,21,22].

It has been found that the structural similarity of FBs with the sphingoid bases sphinganine (Sa) and sphingosine (So) is the main cause to the disruption of sphingolipid metabolisms. FB_1_ can inhibit ceramide synthesis by an unsubstituted primary amino group at C2 that is structurally similar to Sa and So. This leads to the accumulation of Sa and So during the process of sphingolipid biosynthesis [23,24,25]. Moreover, the ratio of free sphingoid bases (Sa/So) is found increase in blood, tissues (e.g., lung, liver, and intestine) and in cultured cells [26,27] by the FB_1_ administration. Therefore, inhibition of ceramide synthase by FB_1_ causes cell damage due to the disruption of the membrane integrity [28].

It has been known that FB_1_ also causes the malfunction of folate transporter and neural tube defects (NTDs), a common congenital abnormality occurs when the embryonic neural tube fails to properly close during the first few weeks of mouse development [29]. The mechanism of FB_1_ action in mammals is due to the inhibition of ceramide synthesis. FB_1_ causes the interruption or aberrant acylation of Sa in the endoplasmic reticulum, the major site of membrane lipid biosynthesis in mammalian cells; in turn, the reduced synthesis of sphingomyelin compromises the proper function of glycosylphosphatidylinositol (GPI)-anchored proteins, such as the folate transporter [30,31] on cell membranes. However, the molecular and cellular mechanisms of FB_1_ action on, e.g., NTDs, in avian species remain to be determined.

Therefore, the present study was aimed to use an in ovo model system to assess the related gene expression profiles and development toxicity of duck embryos after the treatments of various FB_1_ doses. The possible molecular mechanism of FB_1_ during the development of the nervous system in duck embryos was investigated.

## 2. Results

### 2.1. Developmental Toxicity of FB_1_ in Brown Tsaiya Duck (BTD) Embryos

Treatments with FB_1_ did not cause embryo death at 72 h post-incubation (Table 1). Results showed that the viability of embryos was unaffected by the treatment of FB_1_. However, FB_1_ caused a growth retardation and delay of the developmental stage, evaluated by embryonic crown-to-tail length (ECTL) and numbers of somites. The percentages of malformation were higher in the FB_1_ treated groups compared to the untreated control (0% vs. 73.7–88.9%; *p* < 0.0001).

### 2.2. FB_1_ Induces Abnormal Neurulation and Somitogenesis during Early Embryogenesis

**Figure 1:**
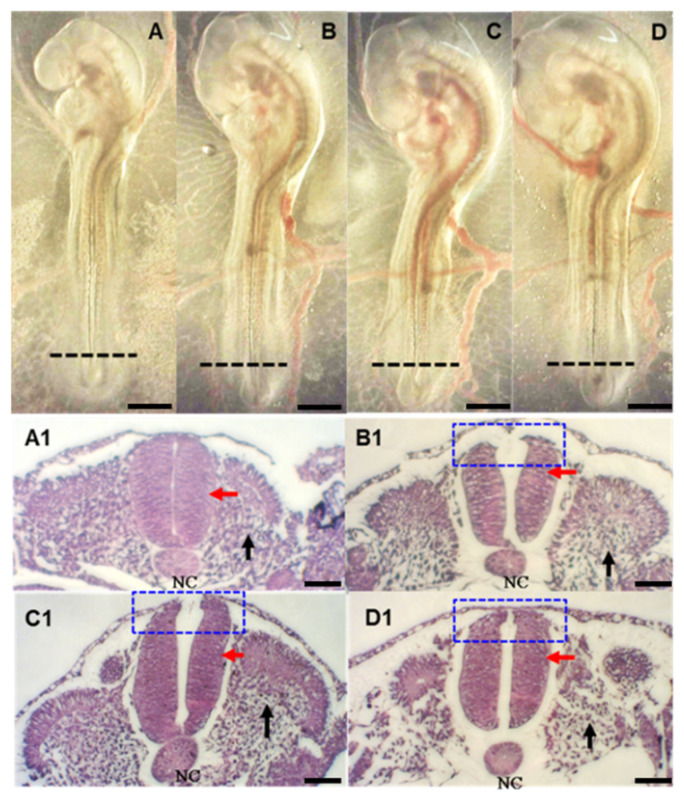
The effect of FB_1_ on neural tube closure of Brown Tsaiya Duck embryos 72 h post-incubation. In the upper panel, (**A**) represents a normal developing embryo without FB_1_ injection (0) and show abnormal images of the neural tube defect after injection with (**B**) (10), (**C**) (20), or (**D**) (40) µg/embryo of FB_1_, respectively. The black dash-line indicates the position of the transverse section. The lower (**A1**–**D1**) panel shows the transverse sections corresponding to (**A**–**D**) in the upper panel, respectively. (**A1**) A normal neural tube closure; (**B1**–**D1**) defective neural tube closure (blue dot box), as well as the cells of the somitomeres appear more edematous or misaligned in comparison with the non-FB_1_ injected control. The red and black arrows indicate the neural tube and somite, respectively. NC: notochord. Scale bar = 0.5 mm.

FB_1_ induced NTDs in BTD embryos are shown in Table 2 (*p* = 0.0417) and Figure 1. The percentages of abnormal neural tube formation and somitogenesis were also significantly increased in FB_1_-treated embryos (*p* < 0.0001) when compared with that in the non-FB_1_ treated control. Morphological examination of the neural tube and the somite by transverse histological sections showed disclosure or defective structures; somitomeres appeared more edematous or misaligned in all groups treated with FB_1_ compared to those in the control (Figure 1).

**Figure 2:**
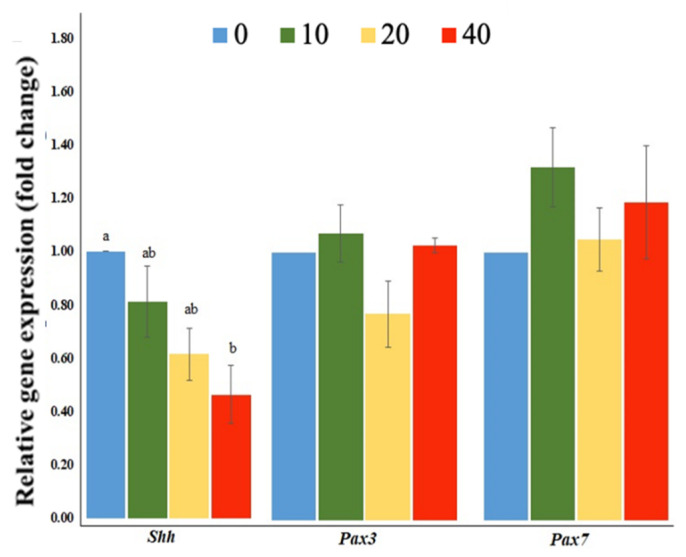
Expressions of the somitogenesis- and neurulation-related genes of Brown Tsaiya Duck embryos with the treatments of FB_1_. Each embryo was injected with 0 (control), 10 (10), 20 (20), or 40 (40) µg of FB_1_, and marker genes including *Shh*, *Pax3*, and *Pax7* are shown. ^a, b^ Columns without the same superscripts differ (*p* < 0.05); three replicates.

The expression of a neurulation-related gene, sonic hedgehog (*Shh*), was down-regulated in the FB_1_-treated group (*p* = 0.0227). However, the marker genes for somite development and neural tube related-genes, Paired Box 3 (*Pax3*) and Paired Box 7 (*Pax7*), were unaffected among different groups (Figure 2).

### 2.3. Exposure to FB_1_ Alters the Sphingolipid Metabolism and the Related-Gene Expression

**Figure 3:**
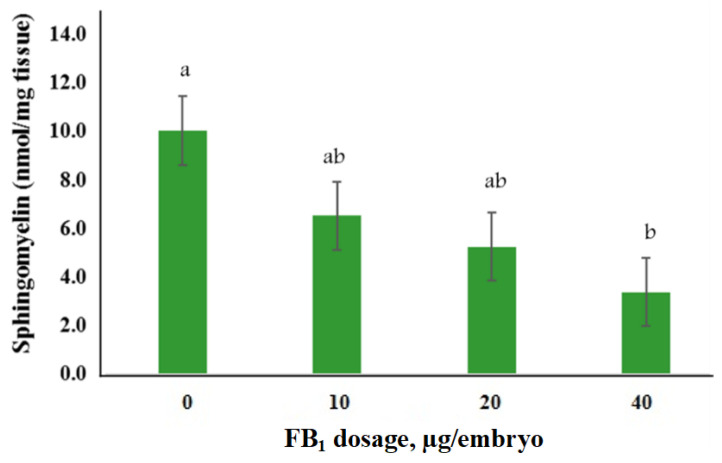
The levels of sphingomyelin in Brown Tsaiya Duck embryonic tissues after injection of various concentrations of FB_1_. ^a, b^ Bars without the same superscripts differ (*p* < 0.05); ten replicates.

The sphingomyelin content was examined for its dose-response relationship with the inhibition of ceramide synthase in sphingolipid metabolism pathway after FB_1_ treatment. Results showed that the sphingomyelin content decreased after the highest dose (40 µg) of FB_1_ treatment in BTD embryos (Figure 3, *p* = 0.0232), when compared with the non-injected control group.

**Figure 4:**
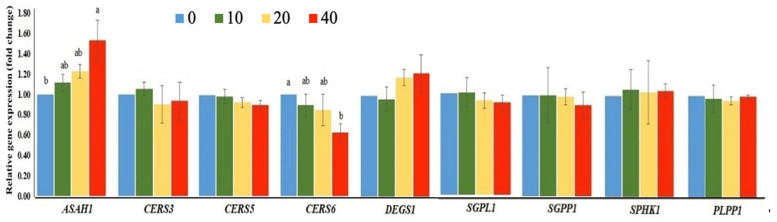
The expression profile of the sphingolipid metabolism-related genes (*ASAH1*: *N*-acylsphingosine amidohydrolase 1; *CERS3*: ceramide synthase 3; *CERS5*: ceramide synthase 5; *CERS6*: ceramide synthase 6; *DEGS1*: delta 4-desaturase, sphingolipid 1; *SGPL1*: sphingosine-1-phosphate lyase 1; *SGPP1*: sphingosine-1-phosphate phosphatase 1; *SPHK1*: sphingosine kinase 1; *PLPP1*: phospholipid phosphatase 1) of Brown Tsaiya Duck (BTD) embryos injected with 0 (control), 10 (10), 20 (20), or 40 (40) µg/embryo of FB_1_. ^a, b^ Bars without the same superscripts differ (*p* < 0.05); three replicates.

**Figure 5:**
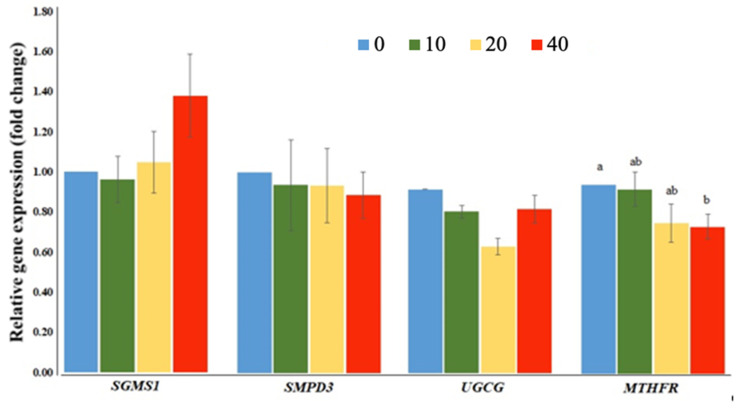
Expressions of sphingomyelin synthase genes (*SMPD3*: sphingomyelin phosphodiesterase 3; *SGMS1*: sphingomyelin synthase 1), glucosylceramide synthase (*UGCG*: UDP-glucose ceramide glucosyltransferase), and folate metabolism-related genes (*MTHFR*: 5,10-methylenetetrahydrofolate reductase) in Brown Tsaiya Duck embryos after FB_1_ injection. Each embryo was injected with 0 (control), 10 (10), 20 (20), or 40 (40) µg of FB_1_. Except for *MTHFR*, no difference among all other treatment groups was detected (*p* > 0.05). ^a, b^ Bars without the same superscripts differ (*p* < 0.05); three replicates.

FB_1_-induced NTDs in BTD embryos could be due to the disturbance of sphingolipid metabolisms. Therefore, we investigated the expression of sphingolipid metabolism-related genes in duck embryonic tissues. Results showed that the sphingolipid metabolism related-genes (*CERS3, CERS5, DEGS1, SGPL1, SGPP1, SPHK1*, and *PLPP1*) (Figure 4), genes coding for sphingomyelin synthase (*SMPD3* and *SGMS1*) and glucosylceramide synthase-related gene; *UGCG* (Figure 5) did not differ among the treatment groups, but N-acylsphingosine amindohydrolase 1 (*ASAH1*) expression was up-regulated in the FB_1_-treated embryos relative to that in the control embryos (*p* = 0.0326). Embryos treated with the highest dose of FB_1_ down-regulated ceramide synthase 6 (*CERS6*) and 5,10-methylenetetrahydrofolate reductase (*MTHFR*) gene expressions compared to those of the control embryos.

## 3. Discussion

**Figure 6:**
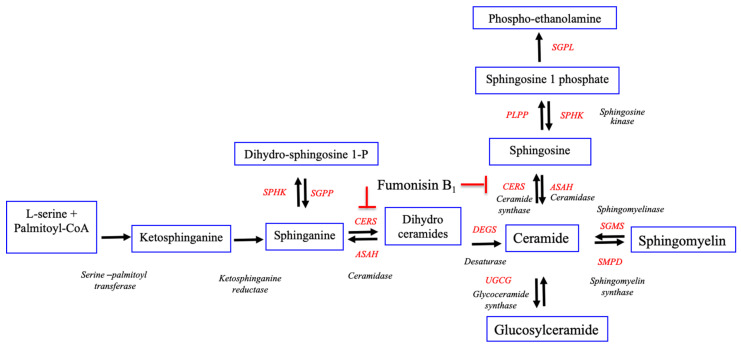
The hypothesis of de novo sphingolipid pathways shows the synthesis of ceramide and sphingomyelin reported by Lumsangkul et al. [33]. The ceramide synthesis pathway could be blocked by FB_1_ indicated by red line.

It has been known that FB_1_ toxicity involves in the disturbance of the sphingolipid metabolisms. In the present study, we found that FB_1_ was a strong inhibitor of ceramide synthase. It disturbed the metabolism of the sphingolipids (Figure 6) which is essential for stabilizing the structure and function of the developing embryos.

The avian embryo is an excellent model system to study the developmental toxicity and mechanisms involving the patterning during early embryogenesis [34]. In previous studies, avian embryos were used to study neurulation, somitogenesis and the effects of various chemicals, such as choline, caffeine, and glucose, on early development of the neural tube and somites [35,36,37,38,39]. In the present study, BTD embryos were used to investigate the toxicity of FB_1_ on the induction of NTDs. The possible molecular mechanism of FB_1_ in relation to the developmental toxicity of BTD embryos was also studied.

Due to little evidence available on the toxic effect of FB_1_ to avian embryogenesis, to our best knowledge, the present study is the first report on the FB_1_ toxicity during early embryogenesis and later development. The FB_1_ intoxicated the BTD embryos by affecting their developmental stages manifested by the growth retardation and consequently the NTDs. The retardation of the embryonic development measured by the ECTL, somite numbers (Table 1), as well as the FB_1_-induced NTDs was confirmed. Our finding is consistent with the observation reported by Liao et al. [40] who demonstrated that FB_1_-induced NTDs of fetuses when a pregnant mouse was fed with FB_1_-contaminated feeds.

Although the NTDs were not discernible in general morphology of the whole embryo (Figure 1A–D), unambiguous NTD structures were observed through the transverse histological sections at the lambo-sacral region or lower trunk (Figure 1B1–D1). Morphological analysis by H&E staining revealed clearly the presence of NTDs in the FB1-treated embryos (Figure 1B1–D1) but not in the control group (Figure 1A,A1). Normally, neural tube closure is a dynamic process that starts from the head region of the neural plate and progresses toward the caudal region, where the two opposing neural folds elevate and fuse together at the midline by epithelial cell adhesion molecule (EpCAM) [41,42]. The fused edge at the dorsal neural tube is smooth and curved; however, embryos exposed to FB_1_ could result in forming a discontinuous dorsal neural tube edge (Figure 1B1–D1). Our observation clearly showed that FB_1_ could disrupt the cellular activity during neurulation and consequently lead to NTDs.

Segmentation is initiated at very early stage in development through the formation of embryonic somites. Somitogenesis plays a very important role in establishing the bone and skeletal muscles in the body and limbs [43], which is closely associated with the neurulation process. It has been shown that the neural tube is required for proper somitogenesis and differentiation [44]. The development and differentiation of somites are dependent on signals emitted from the ipsilateral neural tube [45]. In this study, we also found that numbers of somites decreased (Table 1) and somitomeres appeared more irregularly scattered in FB_1_-treated embryos compared to those in the control embryos (Figure 1B1–D1).

Sonic hedgehog (Shh) protein, an important morphogen, is generated from the noto-chord and the floor plate to regulate many morphogenetic events during early embryo development, such as somitogenesis [46]. It is also involved in myogenesis by inducing myogenic factor (*Myf5*) expression directly and myoblast determination protein (*MyoD*) indirectly [47]. Paired Box 7 (*Pax7*) and Paired Box 3 (*Pax3*) are two key genes expressing in dorsal neural tube and dorsal pre-migratory neural crest cells (NCCs). These genes are expressed in a population of muscle precursor cells to maintain their uncommitted state throughout embryonic development as well as to play a key role in embryonic muscle development [48,49,50]. In the present study, these somitogenesis and neurulation related-genes were interfered along with a decreased expression of *Shh* after FB_1_ treatment (Figure 2).

Due to the structural similarity to sphingosine (So) and sphinganine (Sa), the primary precursors of sphingolipids, FB_1_ could compete with Sa and So for their integration into the sphingolipid metabolism pathway. However, it is unclear why the expressions of sphingolipid metabolism related-genes (*CERS5, SGPL1, SGPP1, SPHK1, and PLPP1*), sphingomyelin synthase (*SMPD3* and *SGMS1*) and glucosylceramide synthase-related genes (*UGCG*) were not affected by FB_1_, which requires more investigation.

To our knowledge, we are the first report to analyze different isoforms of *CERS* gene in avian species. We found that the isoform *CERS6* was related to FB_1_ toxicity, likely, via inhibition of ceramide synthase activity and disturbance of sphingolipids metabolisms in BTDs. As abovementioned, we also showed that FB_1_ increased incidence of NTDs by impairing the functions of folate transporters. Sphingomyelin is a major component of the plasma membrane and is required for the proper function of GPI-anchored proteins of the folate transporter [29]. In this study, we found that the sphingomyelin content in BTD embryo was reduced by FB_1_ treatments (Figure 3) due to the inhibition of sphingolipid biosynthesis. We also found that *MTHFR* gene in folate metabolism pathway was altered in FB_1_-treated BTD embryos. Fumonisin B_1_ might decrease the glycosphingolipids synthesis that in turn increased the incidence of incomplete neurulation or NTDs during embryogenesis.

Ceramidases (*N*-acylsphingosine amidohydrolase, *ASAH1*) are a family of hydrolases that directly regulates the intracellular balance of ceramide by catalyzing the degradation of ceramide into sphingosine. Because ceramide degradation is the only source of sphingosine, these enzymes are not only essential for modulating ceramide-mediated signaling but also for the functions of sphingosine and sphingosine-1-phosphate. In the mouse, *ASAH1* expresses during early embryogenesis and disruption of *ASAH1* gene results in embryonic lethality [51]. In addition, *ASAH1* overexpression has been reported in various human cancers [52]. The sphingosine is a sensitive biomarker for FB_1_ exposure in animals, as well as being proposed for monitoring FB_1_ exposure in humans. It correlates with liver and kidney toxicity and often a precede sign of intoxication [53,54]. In the present study, the highest dose (40 µg) of FB1 exposure up-regulated *ASAH1* expression compared to the control group. Such alterations might be resulted in changes of sphingosine levels and imbalance of ceramide through modulating the expression of *ASAH1* gene. Therefore, embryos showing a disturbance ceramide synthesis profile may be involved in neurodegeneration and reduction of neural cell numbers in the early developing brain [55,56,57], and finally led to NTDs of fetuses.

The metabolism and the mode of action of the FB_1_ in avian species are largely unknown. The present study provided the evidence about the effect of FB_1_ exposure on the levels of ceramide synthase and sphingomyelin. Moreover, FB_1_ might also affect the function of folate transporter and cause NTDs. As abovementioned, folate transporter is a major component of the plasma membrane and is required for the proper function of GPI-anchored proteins. Our observations echo with the findings of Marasas et al. (2004) and Liao et al. (2004) [29,40], who have reported that FB_1_ caused both neural tube and craniofacial defects in mouse embryos by inhibiting sphingolipid biosynthesis, as well as folate transport. Moreover, sphingolipids and cholesterol are typically embedded in the lipids rafts of extracellular leaflet of the cell membrane; interactions between cholesterol, sphingomyelin and FB_1_ warrant further investigation [58,59].

In conclusion, FB_1_ induces growth retardation, developmental abnormalities and neural tube defects of BTD embryos. The underlying mechanism is, at least, partially mediated by disrupting ceramide synthesis in sphingolipid metabolisms.

## 4. Materials and Methods

### 4.1. Eggs Used and Conditions of Incubation

A total of 80 fertilized BTD eggs obtained from Yilan Livestock Research Institute, Council of Agriculture were used in this study. All the eggs were preselected based on weight (61 ± 0.09 g) and randomly assigned into four treatment groups; twenty BTD eggs in each group were incubated in an incubator (YC-M10, Yongcheng Incubation Machine Technology Co., Ltd., Changhua, Taiwan) at 37.5 °C with 65% relative humidity. The whole study was carried out in strict accordance with the guideline recommended and approved by the Institutional Animal Care and Use Committee 365 (IACUC) of the National Chung Hsing University (Permit number: 100–02; date of approval: 24 January 2011).

### 4.2. Treatment Concentrations of Fumonisin B_1_

The FB_1_ was purchased from (Cat No. 116355-83-0, Sigma-Aldrich, St. Louis, MO, USA). The FB_1_ solutions were prepared with deuterium-depleted water (DDW) to a stock concentration of 1 mg/mL (1000 ppm) and diluted into 10, 20 and 40 µg before use.

Various FB_1_ injection dosages (0, 10, 20 and 40 µg/embryo) were directly applied at 0 h post-incubation to embryos in ovo. Briefly, and approximately 100 µL FB_1_ solution was injected into the air chamber of the egg via a small hole made at the blunt-end of the egg. After that, the hole of the egg was sealed with the adhesive tape. The treated embryos were then incubated for 72 h for further observation.

### 4.3. Embryo Viability and Development

Twenty BTD eggs per treatment group were respectively dissected at 72 h post-incubation to determine embryo viability. Embryonic development and viability were defined and distinguished based on the standard speed of developmental progression established previously [32]; parameters including heartbeats, formation of yolk-sac and blood vessels, brain development, as well as organogenesis were examined.

### 4.4. Histological Assessments

After 72 h post-incubation, ten embryos were sampled from each treatment group and were fixed in 10% buffered formalin for 24 h. The tissues of embryos were pre-embedded in standard agarose gel prior to embedding in paraffin wax. Three-micrometer thick sections were cut consecutively from the paraffin blocks, mounted onto glass slides, de-paraffinized, and then dehydrated. Cross sections of the neural tube at the lumbosacral region were stained with hematoxylin and eosin (H&E) to assess morphologies of the neural tube and somite structures. The sections were examined and recorded using a DinoCapture 2.0 digital microscope (Dino-Lite, Los Angeles, CA, USA) attached to a Nikon LABOPHOT-2 binocular microscope (Nikon, Tokyo, Japan).

### 4.5. Sphingomyelin Quantification by Colorimetric Assay

Ten embryos from each group were collected and crushed by ultrasonic cell crusher (40 Hz) followed by the extraction with chloroform and methanol (2:1, *v*/*v*). Liquids were dried in a vacuum dryer and re-dissolved by using tert-Butyl alcohol (TBA). Sphingomyelin levels were analyzed by using sphingomyelin colorimetric Assay Kit (Cayman, MI, USA, Item no: 10009928). Sphingomyelin content was quantified with a Molecular Devices ELISA reader (Sunnyvale, CA, USA) at a wavelength 595 nm based on a sphingomyelin standard curve.

### 4.6. Revers Transcription and Quantitative Real-Time PCR (qPCR) Analyses

Total RNAs were extracted with a total RNA extraction kit (Invitrogen, PureLink™ RNA Mini Kit, Carlsbad, CA, USA). Cytoplasmic RNAs from embryos were reverse-transcribed to generate first-strand cDNA by iScript™ cDNA Synthesis Kit (Bio-Rad, Hercules, CA, USA). Candidate genes (Table 3) were chosen for qPCR analysis. The qPCR was performed in triplicate using 100 ng of cDNA, 0.8 (0.25) mM of primers and iTaq Universal SYBR Green supermix (2X) on a CFX Connect™ Real-Time PCR System (Bio-Rad, Hercules, CA, USA). Thermal cycling conditions were 95 °C for 30 s (holding stage), 40 cycles of 95 °C for 15 s and 60 °C for 30 s (cycling stage) and followed by 95 °C for 15 s, 60 °C for 60 s, 95 °C for 15 s (melt curve stage). The relative expression of genes was analyzed according to the 2^−ΔΔCt^ method. Each sample of each group was measured in triplicate and the assay was repeated three times. The quantification was standardized to an endogenous control GAPDH.

### 4.7. Statistical Analyses

All experimental data were analyzed using analysis of variance (ANOVA) procedure of SAS Enterprise Guide Software V.9.4 (SAS Institute, Cary, NC, USA). Least square means (LSM) were compared by using Tukey’s test. A probability level at *p* < 0.05 was considered as statistically significant.

## Figures and Tables

**Table 1:** Effects of various concentrations of FB_1_ injection on the viability and development of Brown Tsaiya Duck (BTD) embryos 72 h post-incubation.

Parameter	FB_1_ Dosage, µg/Embryo				*p* Value
	0 ^1^	10	20	40	
No. of embryo	18	19	16	18	
Viability, % (n) ^2^	100 (18)	100 (19)	100 (16)	100 (18)	
Embryonic stage ^3^	16–17	16–17	10–17	12–17	
ECTL ^4^, mm	4.43–7.41	5.28–6.54	3.02–8.35	3.08–6.59	
No. of somite	30.3 ± 1.70	29.9 ± 1.73	26.5 ± 6.74	27.9 ± 4.14	
Malformations or delay, % (n) ^5^	0 ^b^	73.7 (14) ^a^	81.3 (13) ^a^	88.9 (16) ^a^	<0.0001

^1^ Non-treated embryos. ^2^ Viability = (No. of live embryos/total of embryos) × 100. ^3^ Embryonic development based on BTD staging system reported by Lumsangkul, et al. [32]. ^4^ Embryonic crown-to-tail length. ^5^ Delayed development is compared with the stage of the control group (0 µg). ^a, b^ Within the row, means without the same superscripts differ (*p* < 0.05).

**Table 2:** Effects of various concentrations of FB_1_ treatment on the development of neural tubes and somites in Brown Tsaiya Duck embryos 72 h post-incubation.

Parameter	FB_1_ Dosage, µg/Embryo				*p* Value
	0 ^1^	10	20	40	
No. of Embryos	10	10	9	8	
Failure of neural tube closure, % (n)	0 ^b^	50 (5) ^a^	55.6(5)^a^	37.5 (3) ^ab^	0.0417
Abnormal neural tube, % (n)	0 (0) ^c^	50 (5) ^b^	100 (9) ^a^	100 (8) ^a^	<0.0001
Abnormal somites, % (n)	10 (1) ^b^	100 (10) ^a^	100 (9) ^a^	100 (8) ^a^	<0.0001
Neural tube width, mm	0.51 ± 0.09	0.51 ± 0.13	0.59 ± 0.20	0.52 ± 0.08	0.4510

^1^ Non-treated embryos. ^a, b, c^ Within the row, means without the same superscripts differ (*p* < 0.05).

**Table 3:** Primer sequences, amplicons and the related information for quantitative real time PCR.

Target Genes	Primers	Sequences (5′/3′)	Product Size	Accession No.
*Sphingolipid metabolism pathway*				
*CERS3*	Forward	GTGCCACGTTGTATCAACCT	172	XM_005011097
	Reverse	TCGCTTCGGTCGTCTTTCAA		
*CERS6*	Forward	CCTTCTGTTCCTTACGTTTGCC	153	XM_038182293
	Reverse	TGAAGAACCACAAGCAACACA		
*CERS5*	Forward	ATCATTCGCACCGCCTACAA	202	XM_021273744
	Reverse	ACCCACAGCCTTACTCCTCT		
*SPHK1*	Forward	ACTGCACCTTCATCCTCTGC	234	XM_027471697
	Reverse	AAAACAGGTAGGAGAGGCGG		
*DEGS1*	Forward	GCACCACGACTTCCCCAATA	155	XM_027453068
	Reverse	TCATGCGTGAATATGGGCTGA		
*SGPL1*	Forward	TTCCCTTCCACGTTGATGCC	212	XM_013107606
	Reverse	GGGTGCCACAAAGAACTGGT		
*SGPP1*	Forward	TGCTGGTGTTTATTGGTTTGCT	205	XM_005022680
	Reverse	TGAAAGAGAAGATGCCCAGGG		
*ASAH1*	Forward	AGGATGCAAAAGACAAACTGGC	158	XM_038178632
	Reverse	CACATACCACGTGCCCTTCT		
*PLPP1*	Forward	TGTAGTGACGAATCCATCCAGT	248	XM_038169951
	Reverse	GCATACTTGGCAATGTCCGTC		
*Sphingomyelin synthase*				
*SMPD3*	Forward	GGTCTACAGTTGCCATGCCT	182	XM_021276493
	Reverse	GGTCCTGAGGTGTACTTCCC		
*SGMS1*	Forward	ATCACTGGCTTTGCTGGACA	194	XM_005017312
	Reverse	GTACCACCAGACCCTTGCAA		
*Glucosylceramide synthase*				
*UGCG*	Forward	ACAGACAGGGATTTGCTGCT	152	XM_038170717
	Reverse	CAGTCCTCCAGCTTGATCCA		
*Folate metabolism pathway*				
*MTHFR*	Forward	CCTGGCATCTTCCCCATACA	226	XM_013101210
	Reverse	TAGCCACTTCCCGATTGAGG		
*Development of neural tube and somite*				
*Pax3*	Forward	GTCAATCAGCTCGGAGGAGT	166	XM_038183383
	Reverse	TCTCCTGGTACCTGCAGAGA		
*Pax7*	Forward	GAGTTCAGGTGTGGTTCAGCA	169	NM_001310395
	Reverse	GAAATGGTGGTGGTTGGGTAG		
*Shh*	Forward	AGGAGTCGCTGCATTACGAG	250	XM_038175308
	Reverse	CTCAGGTCCTTCACCAGCTT		
*Housekeeping gene*				
*GAPDH*	Forward	CTGGCATTGCACTGAACGAC	165	XM_038180584
	Reverse	CTCCAACAAAGGGTCCTGCT		

*CERS3*: ceramide synthase 3; *CERS5*: ceramide synthase 5; *CERS6*: ceramide synthase 6; *DEGS1*: delta 4-desaturase,.sphingolipid 1; *SGPL1*: sphingosine-1-phosphate lyase 1; *SGPP1*: sphingosine-1-phosphate phosphatase 1; *SPHK1*: sphingosine kinase 1; *ASAH1*: *N*-acylsphingosine amidohydrolase 1; *PLPP1*: phospholipid phosphatase 1; *SMPD3*: sphingomyelin phosphodiesterase 3; *SGMS1*: sphingomyelin synthase 1; *UGCG*: UDP-glucose ceramide glucosyltrans ferase; *MTHFR*: 5,10-methylenetetrahydrofolate reductase; *Shh*: sonic hedgehog; *Pax3*: Paired Box 3; *Pax7*: Paired Box 7.

## Data Availability

Data available in a publicly accessible repository.

## References

[1] Bezuidenhout S.C., Gelderblom W.C., Gorst-Allman C.P., Horak R.M., Marasas W.F., Spiteller G., Vleggaar R. (1988). Structure elucidation of the fumonisins, mycotoxins from *Fusarium* moniliforme. J. Chem. Soc. Chem. Commun..

[2] Gelderblom W., Jaskiewicz K., Marasas W., Thiel P., Horak R., Vleggaar R., Kriek N. (1988). *Fumonisins*—Novel mycotoxins with cancer-promoting activity produced by *Fusarium moniliforme*. Appl. Environ. Microbiol..

[3] Anfossi L., Giovannoli C., Baggiani C. (2016). Mycotoxin detection. Curr. Opin. Biotechnol..

[4] Commission Regulation (2007). Commission Regulation (EC) No. 1126/2007 of 28 September 2007 Amending Regulation (EC) No. 1881/2006 Setting Maximum Levels for Certain Contaminants in Foodstuffs as Regards *Fusarium* Toxins in Maize and Maize Products. Off. J. Eur. Union.

[5] El Darra N., Gambacorta L., Solfrizzo M. (2019). Multimycotoxins occurrence in spices and herbs commercialized in Lebanon. Food Control.

[6] Mendes G.D.R.L., Reis T.A.D., Corrêa B., Badiale-Furlong E. (2015). Mycobiota and occurrence of *Fumonisin* B_1_ in wheat harvested in Southern Brazil. Ciência. Rural..

[7] Thiel P.G., Shephard G.S., Sydenham E.W., Marasas W.F., Nelson P.E., Wilson T.M. (1991). Levels of fumonisins B1 and B2 in feeds associated with confirmed cases of equine leukoencephalomalacia. J. Agric. Food Chem..

[8] Shephard G.S., Thiel P.G., Stockenström S., Sydenham E.W. (1996). Worldwide survey of fumonisin contamination of corn and corn-based products. J. AOAC Int..

[9] Howard P.C., Couch L.H., Patton R.E., Eppley R.M., Doerge D.R., Churchwell M.I., Marques M.M., Okerberg C.V. (2002). Comparison of the toxicity of several fumonisin derivatives in a 28-day feeding study with female B6C3F1 mice. Toxicol. Appl. Pharmacol..

[10] Buszewska-Forajta M. (2020). Mycotoxins, invisible danger of feedstuff with toxic effect on animals. Toxicon.

[11] Marasas W.F. (1996). *Fumonisins*: History, world-wide occurrence and impact. Fumonisins Food.

[12] Rheeder J.P., Marasas W.F., Thiel P.G., Sydenham E.W., Shephard G.S., Van Schalkwyk D.J. (1992). *Fusarium moniliforme* and fumonisins in corn in relation to human esophageal cancer in Transkei. Phytopathology. Postharvest Pathol. Mycotoxins.

[13] Van der Westhuizen L., Shephard G.S., Rheeder J., Burger H.-M. (2010). Individual fumonisin exposure and sphingoid base levels in rural populations consuming maize in South Africa. Food Chem. Toxicol..

[14] Marasas W.F.O., Kellerman T.S., Gelderblom W.C., Thiel P., Van der Lugt J.J., Coetzer J.A. (1988). Leukoencephalomalacia in a horse induced by fumonisin B_1_ isolated from *Fusarium moniliforme*. Onderstepoort J. Vet. Res..

[15] Colvin B.M., Harrison L.R. (1992). *Fumonisin*-induced pulmonary edema and hydrothorax in swine. Mycopathologia.

[16] Voss K., Smith G., Haschek W. (2007). *Fumonisins*: Toxicokinetics, mechanism of action and toxicity. Anim. Feed Sci. Technol..

[17] Voss K.A., Chamberlain W.J., Bacon C.W., Norred W.P. (1993). A preliminary investigation on renal and hepatic toxicity in rats fed purified fumonisin B_1_. Nat. Toxins..

[18] Voss K.A., Riley R.T., Norred W., Bacon C.W., Meredith F.I., Howard P.C., Plattner R.D., Collins T., Hansen D.K., Porter J.K. (2001). An overview of rodent toxicities: Liver and kidney effects of fumonisins and *Fusarium moniliforme*. Environ. Health Perspect..

[19] Bouhet S., Oswald I.P. (2007). The intestine as a possible target for fumonisin toxicity. Mol. Nutr. Food Res..

[20] Bouhet S., Hourcade E., Loiseau N., Fikry A., Martinez S., Roselli M., Galtier P., Mengheri E., Oswald I.P. (2004). The mycotoxin fumonisin B1 alters the proliferation and the barrier function of porcine intestinal epithelial cells. Toxicol. Sci..

[21] Loiseau N., Polizzi A., Dupuy A., Therville N., Rakotonirainy M., Loy J., Viadere J.-L., Cossalter A.-M., Bailly J.-D., Puel O. (2015). New insights into the organ-specific adverse effects of fumonisin B1: Comparison between lung and liver. Arch. Toxicol..

[22] Smith G.W., Constable P.D., Eppley R.M., Tumbleson M.E., Gumprecht L.A., Haschek-Hock W.M. (2000). Purified fumonisin B1 decreases cardiovascular function but does not alter pulmonary capillary permeability in swine. Toxicol. Sci..

[23] Wang E., Norred W., Bacon C., Riley R., Merrill A.H. (1991). Inhibition of sphingolipid biosynthesis by fumonisins. Implications for diseases associated with *Fusarium moniliforme*. J. Biol. Chem..

[24] Wang E., Ross P.F., Wilson T.M., Riley R.T., Merrill A.H. (1992). Increases in serum sphingosine and sphinganine and decreases in complex sphingolipids in ponies given feed containing fumonisins, mycotoxins produced by *Fusarium moniliforme*. J. Nutr..

[25] Merrill Jr A.H., Sullards M.C., Wang E., Voss K.A., Riley R.T. (2001). Sphingolipid metabolism: Roles in signal transduction and disruption by fumonisins. Environ. Health Perspect..

[26] Riley R.T., An N.-H., Showker J.L., Yoo H.-S., Norred W.P., Chamberlain W.J., Wang E., Merrill A.H., Motelin G., Beasley V.R. (1993). Alteration of tissue and serum sphinganine to sphingosine ratio: An early biomarker of exposure to fumonisin-containing feeds in pigs. Toxicol. Appl. pharmacol..

[27] Loiseau N., Debrauwer L., Sambou T., Bouhet S., Miller J.D., Martin P.G., Viadère J.-L., Pinton P., Puel O., Pineau T. (2007). *Fumonisin* B_1_ exposure and its selective effect on porcine jejunal segment: Sphingolipids, glycolipids and trans-epithelial passage disturbance. Biochem. Pharmacol..

[28] Riley R.T., Enongene E., Voss K.A., Norred W.P., Meredith F.I., Sharma R.P., Spitsbergen J., Williams D.E., Carlson D.B., Merrill A.H. (2001). Sphingolipid perturbations as mechanisms for fumonisin carcinogenesis. Environ. Health Perspect..

[29] Marasas W.F., Riley R.T., Hendricks K.A., Stevens V.L., Sadler T.W., Gelineau-van Waes J., Missmer S.A., Cabrera J., Torres O., Gelderblom W.C. (2004). *Fumonisins* disrupt sphingolipid metabolism, folate transport, and neural tube development in embryo culture and in vivo: A potential risk factor for human neural tube defects among populations consuming fumonisin-contaminated maize. J. Nutr..

[30] Tidhar R., Futerman A.H. (2013). The complexity of sphingolipid biosynthesis in the endoplasmic reticulum. Biochim. Biophys. Acta Mol. Cell Res..

[31] Jan J.-T., Chatterjee S., Griffin D.E. (2000). Sindbis virus entry into cells triggers apoptosis by activating sphingomyelinase, leading to the release of ceramide. J. Virol..

[32] Lumsangkul C., Fan Y.-K., Chang S.-C., Ju J.-C., Chiang H.-I. (2018). Characterizing early embryonic development of Brown Tsaiya Ducks (*Anas platyrhynchos*) in comparison with Taiwan Country Chicken (*Gallus gallus domestics*). PLoS ONE.

[33] Lumsangkul C., Chiang H.-I., Lo N.-W., Fan Y.-K., Ju J.-C. (2019). Developmental toxicity of mycotoxin *Fumonisin* B_1_ in animal embryogenesis: An overview. Toxins.

[34] Tufan A.C., Akdogan I., Adiguzel E. (2004). Shell-less culture of the chick embryo as a model system in the study of developmental neurobiology. Neuroanatomy.

[35] Song G., Cui Y., Han Z.-J., Xia H.-F., Ma X. (2012). Effects of choline on sodium arsenite-induced neural tube defects in chick embryos. Food Chem. Toxicol..

[36] Ma Z.-L., Qin Y., Wang G., Li X.-D., He R.-R., Chuai M., Kurihara H., Yang X. (2012). Exploring the caffeine-induced teratogenicity on neurodevelopment using early chick embryo. PLoS ONE.

[37] Chen Y., Fan J.-X., Zhang Z.-L., Wang G., Cheng X., Chuai M., Lee K.K.H., Yang X. (2013). The negative influence of high-glucose ambience on neurogenesis in developing quail embryos. PLoS ONE.

[38] Chen Y., Wang G., Ma Z.-L., Li Y., Wang X.-Y., Cheng X., Chuai M., Tang S.-Z., Lee K.K.H., Yang X. (2014). Adverse effects of high glucose levels on somite and limb development in avian embryos. Food Chem. Toxicol..

[39] Cheng X., Wang G., Ma Z.-L., Chen Y.-Y., Fan J.-J., Zhang Z.-L., Lee K.K.H., Luo H.-M., Yang X. (2012). Exposure to 2, 5-hexanedione can induce neural malformations in chick embryos. Neurotoxicology.

[40] Liao Y.-J., Yang J.-R., Chen S.-E., Wu S.-J., Huang S.-Y., Lin J.-J., Chen L.-R., Tang P.-C. (2014). Inhibition of fumonisin B1 cytotoxicity by nanosilicate platelets during mouse embryo development. PLoS ONE.

[41] Nikolopoulou E., Galea G.L., Rolo A., Greene N.D., Copp A.J. (2017). Neural tube closure: Cellular, molecular and biomechanical mechanisms. Development.

[42] Greene N.D., Copp A.J. (2014). Neural tube defects. Annu. Rev. Neurosci..

[43] Dequéant M.-L., Pourquié O. (2008). Segmental patterning of the vertebrate embryonic axis. Nat. Rev. Genet..

[44] Wang G., Li Y., Wang X.-Y., Chuai M., Yeuk-Hon Chan J., Lei J., Münsterberg A., Lee K.K.H., Yang X. (2015). Misexpression of BRE gene in the developing chick neural tube affects neurulation and somitogenesis. Mol. Biol. Cell..

[45] Sela-Donenfeld D., Kalcheim C. (2000). Inhibition of noggin expression in the dorsal neural tube by somitogenesis: A mechanism for coordinating the timing of neural crest emigration. Development.

[46] Resende T.P., Ferreira M., Teillet M.-A., Tavares A.T., Andrade R.P., Palmeirim I. (2010). Sonic hedgehog in temporal control of somite formation. Proc. Nati. Acad. Sci. USA.

[47] Chiang C., Litingtung Y., Lee E., Young K.E., Corden J.L., Westphal H., Beachy P.A. (1996). Cyclopia and defective axial patterning in mice lacking Sonic hedgehog gene function. Nature.

[48] Otto A., Schmidt C., Patel K. (2006). Pax3 and Pax7 expression and regulation in the avian embryo. Anat. Embryol..

[49] Galli L.M., Knight S.R., Barnes T.L., Doak A.K., Kadzik R.S., Burrus L.W. (2008). Identification and characterization of subpopulations of Pax3 and Pax7 expressing cells in developing chick somites and limb buds. Dev. Dyn..

[50] Relaix F., Rocancourt D., Mansouri A., Buckingham M. (2004). Divergent functions of murine Pax3 and Pax7 in limb muscle development. Genes Dev..

[51] Eliyahu E., Park J.-H., Shtraizent N., He X., Schuchman E.H. (2007). Acid ceramidase is a novel factor required for early embryo survival. FASEB J..

[52] Saad A.F., Meacham W.D., Bai A., Anelli V., Anelli V., Mahdy A.E., Turner L.S., Cheng J., Bielawska A., Bielawski J. (2007). The functional effects of acid ceramidase over-expression in prostate cancer progression and resistance to chemotherapy. Cancer Biol. Ther..

[53] Tran S.T., Tardieu D., Auvergne A., Bailly J.D., Babilé R., Durand S., Benard G., Guerre P. (2006). Serum sphinganine and the sphinganine to sphingosine ratio as a biomarker of dietary fumonisins during chronic exposure in ducks. Chem. Biol. Interact..

[54] Tardieu D., Bailly J.-D., Benlashehr I., Auby A., Jouglar J.-Y., Guerre P. (2009). Tissue persistence of fumonisin B1 in ducks and after exposure to a diet containing the maximum European tolerance for fumonisins in avian feeds. Chem. Biol. Interact..

[55] Ben-David O., Futerman A.H. (2010). The role of the ceramide acyl chain length in neurodegeneration: Involvement of ceramide synthases. Neuromol. Med..

[56] Riley R.T., Merrill A.H. (2019). Ceramide synthase inhibition by fumonisins: A perfect storm of perturbed sphingolipid metabolism, signaling, and disease. J. Lipid Res..

[57] Spassieva S.D., Ji X., Liu Y., Gable K., Bielawski J., Dunn T.M., Bieberich E., Zhao L. (2016). Ectopic expression of ceramide synthase 2 in neurons suppresses neurodegeneration induced by ceramide synthase 1 deficiency. Proc. Nat. Acad. Sci. USA.

[58] Mahfoud R., Maresca M., Santelli M., Pfohl-Leszkowicz A., Puigserver A., Fantini J. (2002). pH-dependent interaction of fumonisin B1 with cholesterol: Physicochemical and molecular modeling studies at the air− water interface. J. Agric. Food Chem..

[59] Rog T., Vattulainen I. (2014). Cholesterol, sphingolipids, and glycolipids: What do we know about their role in raft-like membranes?. Chem. Phys. Lipids..

